# Characteristics of falls among older hip fracture patients from six Chinese hospitals: a *post-hoc* descriptive analysis

**DOI:** 10.1186/s12877-023-03971-6

**Published:** 2023-05-11

**Authors:** Junyi Peng, Pengpeng Ye, Jing Zhang, Xinyi Zhang, Ke Peng, Jiusheng He, Liangyuan Wen, Xianhai Wang, Zongxin Shi, Sanbao Hu, Fengpo Sun, Zishun Gong, Mingyao Sun, Tingzhuo Liu, Xinyan Liu, Ruofei Ma, Shiwen Zhu, Xinbao Wu, Rebecca Ivers, Minghui Yang, Maoyi Tian

**Affiliations:** 1 School of Public Health, Harbin Medical University, 157 Baojian Road, Nangang District, Harbin, 150081 China; 2 National Centre for Non-Communicable Disease Control and Prevention, Chinese Centre for Disease Control and Prevention, Beijing, China; 3 School of Population Health, Faculty of Medicine and Health, University of New South Wales, Sydney, Australia; 4 National Clinical Research Centre for Cardiovascular Diseases, Fuwai Hospital Chinese Academy of Medical Sciences, Shenzhen, China; 5 Department of Orthopaedics, Beijing Shunyi District Hospital, Beijing, China; 6 Department of Orthopaedics, Beijing Hospital, Institute of Geriatric Medicine, National Centre of Gerontology, Chinese Academy of Medical Sciences, Beijing, China; 7 Department of Orthopaedics, Beijing Changping District Hospital, Beijing, China; 8 Department of Orthopaedics, Beijing Liangxiang Hospital, Beijing, China; 9 Department of Orthopaedics, Beijing Anzhen Hospital, Capital Medical University, Beijing, China; 10 Department of Orthopaedics and Traumatology, Beijing Jishuitan Hospital, Peking University Fourth School of Clinical Medicine, 31 Xinjiekou E Street, Xicheng District, Beijing, 100035 China; 11 The George Institute for Global Health, Faculty of Medicine and Health, University of New South Wales, Sydney, Australia

**Keywords:** Fall-related hip fracture, Characteristics of falls, Epidemiology, Older people, China

## Abstract

**Background:**

There is well-established evidence to understand the characteristics of falls among the older patients with hip fracture in many countries, but very little knowledge existed in China. This study described the characteristics of falls in older patients with hip fractures from six Chinese hospitals.

**Methods:**

This cross-sectional study is a *post-hoc* descriptive analysis of a recently completed trial. Eligible patients were aged 65 years and older, with confirmed hip fractures due to falls, and were admitted to the hospital within 21 days of the fracture. All patients were consecutively enrolled and screened within one year (November 15, 2018, to November 14, 2019). The collected data included patient demographics and fall-related information.

**Results:**

A total of 1,892 patients’ fall-related information were described. Most patients with hip fractures caused by falls were in the oldest old age group (60.4% in age group ≥ 80), with an overall average age of 80.7 (7.6) years. There were more females (n = 1,325, 70.0%) than males (n = 567, 30.0%). The majority lived in urban (n = 1,409, 74.5%). Most falls (n = 1,237, 67.3%) occurred during the daytime (6:01–18:00). There were 1,451 patients had their falls occurring at home (76.7%). Lost balance (n = 1,031, 54.5%) was reported as the primary reason to cause falls. The most common activity during a fall was walking (n = 1,079, 57.0%).

**Conclusions:**

Although the incidence of fall-related hip fractures in China is unclear, preventing falls and fall-related hip fractures in older people remains an urgent health concern as the ageing society increases. Studies with larger sample size and diverse population are needed to robustly understand this growing epidemic.

## Background

Falls are events that result in a person returning to rest inadvertently on the ground, floor, or other lower level, as defined by the World Health Organisation [1]. Globally, more than 684,000 people die from falls each year [1]. Falls are the third leading cause of death resulting from unintentional injury [2]. In particular, they are a major cause of fatal and non-fatal injuries among the older people [3, 4]. Approximately one-third of people aged 65 and older worldwide fall at least once yearly, with three quarters of fatal falls occur in low- and middle-income countries [1]. A single fall can lead to serious consequences for older people, like hip fracture [5, 6]. Over 90% of hip fractures are caused by falls, [7, 8] and is often associated with increased mortality, severe disability, extended hospital stays, and higher medical costs [9, 10].

In China, the number of people aged 65 and older is expected to exceed 300 million in 2033, accounting for 20% of the entire population [11]. With such rapid growth of ageing population, [12] falls have become an important public health issue. A recent Global Burden of Diseases study, described the burden of falls among older people in China over the period from 1990 to 2019, found the incidence rate of falls increased substantially in older people [13]. With implementation of effective falls prevention programs, many countries saw a decline in the incidence of fall-related hip fracture [14, 15]. But in China, scalable high-quality falls prevention interventions are extremely limited, and older people may still experience an increased risk of fall-related hip fracture, with a double burden in ageing and falls.

Despite well-established evidence to understand the characteristics of falls among the older patients with hip fracture in many countries, mostly in high-income countries, [14–16] very little knowledge existed in China [8, 17]. In the previous literature, there was only one study from China describing the location and timing of falls in a small number of older hip fracture patients [17]. Thus, in this study, we aim to conduct a *post-hoc* descriptive analysis using the baseline information from a completed pragmatic trial conducted in six Chinese hospitals, to comprehensively characterise falls among thousands of fall-induced, clinically confirmed older hip fracture patients.

## Methods

### The main trial

A multicentre, non-randomised, controlled trial was conducted in six Chinese hospitals, and recruited a total of 2,071 hip fracture patients aged 65 or above, with X-ray confirmed hip fracture and admitted to hospital within 21 days of injury. This trial sought to assess the effect of a multidisciplinary orthogeriatric co-management program on quality standards of hip fracture management and patients’ clinical outcomes. Findings showed that the orthogeriatric co-management program, implemented at one single urban tertiary hospital, substantially improved the quality of hip fracture care, with shortened admission to surgery time and reduced patients’ one-year mortality [18]. At the baseline, all enrolled patients completed a baseline assessment, including patients’ demographic information, pre-operative, peri-operative and post-operative information. For those patients having a hip fracture due to a fall, the characteristics of the fall was also collected. Details of the trial and its main findings were described elsewhere [18].

### Study design and settings

This study is a *post-hoc* descriptive analysis based on the prior trial, using the baseline cross-sectional information of those hip fracture patients due to a fall. The study was conducted at six hospitals in Beijing, China (three urban hospitals: Beijing Jishuitan Hospital, Beijing Hospital, and Anzhen Hospital; three district suburban hospitals: Beijing Changping District Hospital, Beijing Shunyi District Hospital, and Beijing Liangxiang Hospital). Beijing Jishuitan Hospital implemented the multidisciplinary orthogeriatric co-management intervention, whereas all other hospitals continued the orthopaedics-led usual care. All methods were carried out in accordance with the STrengthening the Reporting of OBservational studies in Epidemiology (STROBE) and the Helsinki Declaration [19].

### Study population

All patients were consecutively enrolled and screened within one year (15 November 2018 to 14 November 2019). We included patients 65 years of age and older with a radiographically diagnosed hip fracture within 21 days of fracture, [20] while patients with pathological (neoplastic) fractures, periprosthetic fractures, advanced malignancies, [18] and patients with hip fractures due to non-falls were excluded. Written informed consent was obtained from all participants.

### Data collection and statistical analysis

We collected information including patient demographics (i.e., age, gender, independent living, living location, education, assistive device, sleeping problem and urinate in night) and fall-related information (i.e., the cause of the falling, falling location, activities when falls occurred, falling direction, time of falling, and if falling at home) using a questionnaire. Trained nurses from the orthopaedic ward in each hospital were responsible for patients’ screening, enrolment, and data collection. Patients with dementia or any difficulties to communicate with the nurse, fall-related information was collected from patients’ family members or their care givers. Continuous and categorical variables are described using means (standard deviations) and proportions respectively. Independent sample t-test was used for continuous variables, while Chi-squared test was used for categorical variables. Fisher’s exact test was used for categorical variables when the expected count was less than five. Statistical Product and Service Solutions (SPSS) software (version 26.0) is used for analysis.

**Fig. 1:**
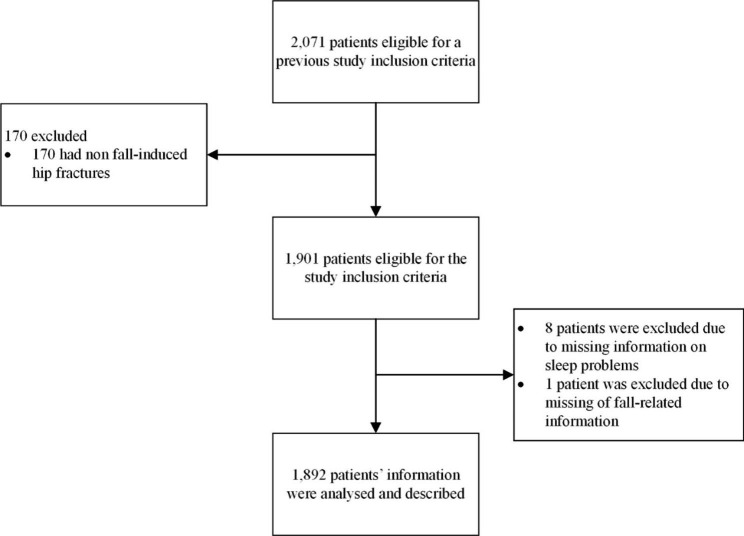
Study flow chart

## Results

A total of 2,071 patients were enrolled into the trial. In this *post-hoc* analysis, 170 patients, who had non-fall induced hip fractures, were excluded. Another nine patients were excluded from the analysis due to the missing information of their baseline or fall-related characteristics. Finally, 1,892 patients’ fall-related information were described. (Fig. 1)

Most patients with hip fractures caused by falls were in the oldest old age group (60.4% in age group ≥ 80), with an overall average age of 80.7 (7.6) years. There were more females (n = 1,325, 70.0%) than males (n = 567, 30.0%). The majority lived in urban (n = 1,409, 74.5%), and there were very few older people living alone (n = 211, 11.2%). Most patients received education from junior high school or below (n = 1,474, 77.8%), with only 10.0% had a degree from university or above. More than half of the patients had self-reported sleeping problems (n = 1,033, 54.6%) and urinated at night (n = 1,414, 74.7%). (Table 1)

**Table 1:** Demographic characteristics of study participants

Characteristic	Female (N = 1,325)	Male (N = 567)	Total (N = 1,892)	P value
Age (years), Mean ± SD	80.6 ± 7.4	80.7 ± 8.0	80.7 ± 7.6	0.038
Age group, n (%)				0.061
65–69	130 (9.8)	75 (13.2)	205 (10.8)	
70–74	171 (12.9)	62 (10.9)	233 (12.3)	
75–79	230 (17.4)	81 (14.3)	311 (16.4)	
80–84	360 (27.2)	147 (25.9)	507 (26.8)	
≥ 85	434 (32.8)	202 (35.6)	636 (33.6)	
Independent living, n (%)	153 (11.5)	58 (10.2)	211 (11.2)	0.404
Living in urban areas, n (%)	1,004 (75.8)	405 (71.4)	1,409 (74.5)	0.047
Education, n (%)				< 0.001
No education	339 (25.6)	87 (15.3)	426 (22.5)	
Primary school or lower	399 (30.1)	179 (31.6)	578 (30.5)	
Junior high school	308 (23.2)	162 (28.6)	470 (24.8)	
Senior high school or technical secondary school	161 (12.2)	68 (12.0)	229 (12.1)	
University or higher	118 (8.9)	71 (12.5)	189 (10.0)	
Using assistive device, n (%)	508 (38.3)	231 (40.7)	739 (39.1)	0.327
Having sleeping problem, n (%)	728 (54.9)	305 (53.8)	1,033 (54.6)	0.645
Urinate in night, n (%)	978 (73.8)	436 (76.9)	1,414 (74.7)	0.157

**Fig. 2:**
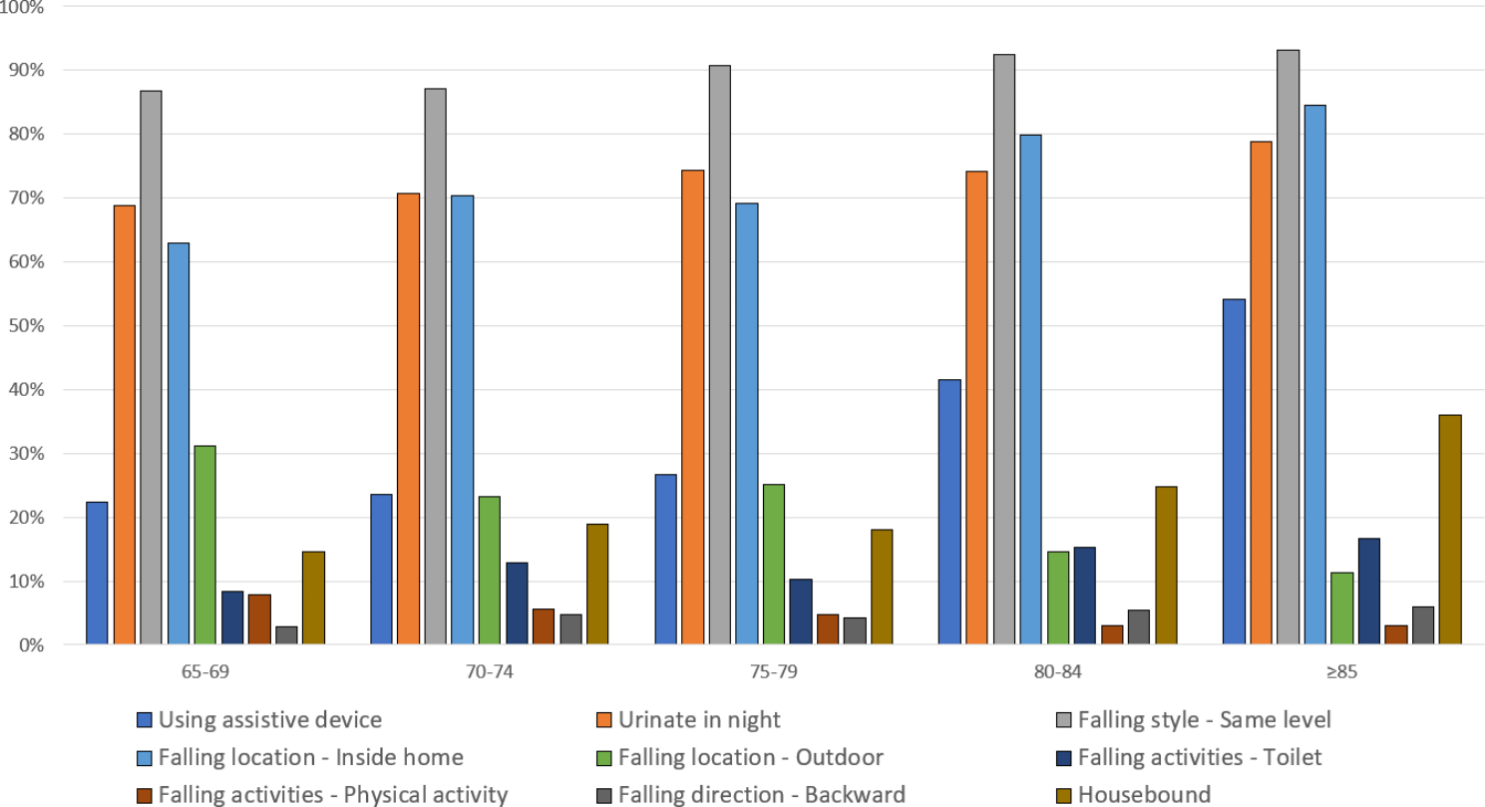
Selected characteristics of the study participants in different age groups

The oldest old people are likely to use assistive devices in daily activities (22.4% in the age group 65–69 to 54.1% in the age group ≥ 85), with an overall usage rate of 39.1%. The proportion of falls at home was higher in the older age groups than in the younger age groups (62.9% in the age group 65–69 to 84.6% in the age group ≥ 85), whereas there was a higher proportion of people from the younger age group fell outdoors (31.2% in the age group 65–69 to 11.3% in the age group ≥ 85). Moreover, more patients in the older age group were housebound. (Fig. 2).

Among 1,892 hip fracture patients, there were 1,021 patients from Beijing Jishuitan Hospital, accounting for 54.0% of the entire participants, while Beijing Anzhen Hospital had the least number of patients enrolled (n = 77, 4.1%). There were more female patients from Beijing Jishuitan Hospital (73.2% versus 66.4% for other hospitals). Patients from Beijing Jishiutan Hospital more likely lived in urban areas (92.2% versus 53.7%) and received higher education compared to other hospitals (14.5% versus 4.7%). (Table 2)

**Table 2:** Demographic characteristics of patients with fall-induced hip fractures across all study sites

Characteristic	Co-management program	Orthopedics-led usual care						P value*
	JST (N = 1021)	BJ (N = 199)	AZ (N = 77)	CP (N = 174)	SY (N = 259)	LX (N = 162)	Subtotal (N = 871)	
Age (years), Mean ± SD	80.4 ± 7.8	82.8 ± 7.4	81.3 ± 6.6	79.8 ± 7.3	80.5 ± 7.4	80.2 ± 7.3	80.9 ± 7.4	0.083
Age group, n (%)								0.070
65–69	130 (12.7)	12 (6.0)	3 (3.9)	22 (12.6)	25 (9.7)	13 (8.0)	75 (8.6)	
70–74	119 (11.7)	19 (9.5)	10 (13.0)	23 (13.2)	32 (12.4)	30 (18.5)	114 (13.1)	
75–79	164 (16.1)	28 (14.1)	13 (16.9)	23 (13.2)	52 (20.1)	31 (19.1)	147 (16.9)	
80–84	268 (26.2)	52 (26.1)	27 (35.1)	53 (30.5)	66 (25.5)	41 (25.3)	239 (27.4)	
≥ 85	340 (33.3)	88 (44.2)	24 (31.2)	53 (30.5)	84 (32.4)	47 (29.0)	296 (34.0)	
Female, n (%)	747 (73.2)	136 (68.3)	60 (77.9)	100 (57.5)	164 (63.3)	118 (72.8)	578 (66.4)	0.001
Independent living, n (%)	122 (11.9)	26 (13.1)	3 (3.9)	21 (12.1)	25 (9.7)	14 (8.6)	89 (10.2)	0.233
Living in urban areas, n (%)	941 (92.2)	193 (97.0)	72 (93.5)	61 (35.1)	68 (26.3)	74 (45.7)	468 (53.7)	< 0.001
Education, n (%)								< 0.001
No education	185 (18.1)	37 (18.6)	13 (16.9)	56 (32.2)	88 (34.0)	47 (29.0)	241 (27.7)	
Primary school or lower	266 (26.1)	29 (14.6)	29 (37.7)	72 (41.4)	118 (45.6)	64 (39.5)	312 (35.8)	
Junior high school	265 (26.0)	64 (32.2)	15 (19.5)	36 (20.7)	49 (18.9)	41 (25.3)	205 (23.5)	
Senior high school or technical secondary school	157 (15.4)	43 (21.6)	10 (13.0)	9 (5.2)	2 (0.8)	8 (4.9)	72 (8.3)	
University or higher	148 (14.5)	26 (13.1)	10 (13.0)	1 (0.6)	2 (0.8)	2 (1.2)	41 (4.7)	
Hip fracture types, n (%)								< 0.001
Neck of femur	518 (50.7)	105 (52.8)	37 (48.1)	53 (30.5)	26 (10.0)	67 (41.4)	288 (33.1)	
Intertrochanteric	480 (47.0)	93 (46.7)	38 (49.4)	117 (67.2)	226 (87.3)	95 (58.6)	569 (65.3)	
Subtrochanteric	23 (2.3)	1 (0.5)	2 (2.6)	4 (2.3)	7 (2.7)	0 (0.0)	14 (1.6)	

*P-values indicate the significant differences in selected characteristics between Beijing Jishuitan Hospital, where the multidisciplinary co-management was implemented and all other hospitals. JST: Beijing Jishuitan Hospital, BJ: Beijing Hospital, AZ: Beijing Anzhen Hospital, CP: Beijing Changping District Hospital, SY: Beijing Shunyi District Hospital, LX: Beijing Liangxiang Hospital

Most falls (n = 1,237, 67.3%) occurred during the daytime (6:01–18:00). The peak time was morning (6:01–12:00) with 749 patients (39.6%) fell. A total of 1,451 patients had their falls occurring at home (76.7%). Female patients (n = 1,029, 77.7%) had a higher rate of falls at home than males (n = 422, 74.4%). There were 342 patients (18.1%) had their falls occurring outdoor. Male patients (n = 199, 21.0%) had a higher rate of falls outdoors than females (n = 223, 16.8%). In addition, 99 patients (5.2%) had their falls occurring in public buildings. Most patients fell at the same level (n = 1,725, 91.2%). Lost balance (n = 1,031, 54.5%) was reported as the primary reason to cause falls, followed by slips (n = 476, 25.2%) and trips (n = 328, 17.3%). The most common activities during falls were walking, followed by toileting, leisure activities, and others. Left (n = 725, 38.3%) and right (n = 768, 40.6%) sidewards were identified as the most common fall directions for patients. (Table 3)

**Table 3:** Characteristics of patients with fall-induced hip fractures between urban and rural areas

Characteristic	Urban (N = 1409)	Rural (N = 483)	Total (N = 1,892)	P value
Soft floor, n (%)	14 (1.0)	4 (0.8)	18 (1.0)	1.000
Housebound, n (%)	361 (25.6)	123 (25.5)	484 (25.6)	0.946
Time of falling, n (%)				0.034
Morning (6:01–12:00)	550 (39.0)	199 (41.2)	749 (39.6)	
Afternoon (12:01–18:00)	378 (26.8)	146 (30.2)	524 (27.7)	
Evening (18:01 − 0:00)	252 (17.9)	85 (17.6)	337 (17.8)	
Night (0:01–6:00)	229 (16.3)	53 (11.0)	282 (14.9)	
Falling style, n (%)				0.389
Same level	1280 (90.8)	445 (92.1)	1725 (91.2)	
From high level to lower level	129 (9.2)	38 (7.9)	167 (8.8)	
Falling cause, n (%)				< 0.001
Only slipped over	318 (22.6)	158 (32.7)	476 (25.2)	
Only tripped over	274 (19.4)	54 (11.2)	328 (17.3)	
Only lost balance (e.g., weakness leg, miss step)	768 (54.5)	263 (54.5)	1031 (54.5)	
Others	8 (0.6)	0 (0.0)	8 (0.4)	
Two or more reasons	41 (2.9)	8 (1.7)	49 (2.6)	
Falling location, n (%)				0.012
Inside home	1058 (75.1)	393 (81.4)	1451 (76.7)	
Public building	75 (5.3)	24 (5.0)	99 (5.2)	
Outdoor	276 (19.6)	66 (13.7)	342 (18.1)	
Falling activities, n (%)				< 0.001
Housework	51 (3.6)	11 (2.3)	62 (3.3)	
Shower	17 (1.2)	9 (1.9)	26 (1.4)	
Toilet	188 (13.3)	74 (15.3)	262 (13.8)	
Walking	792 (56.2)	287 (59.4)	1079 (57.0)	
Physical activity	62 (4.4)	17 (3.5)	79 (4.2)	
Leisure activities (except walking)	166 (11.8)	24 (5.0)	190 (10.0)	
Others	133 (9.4)	61 (12.6)	194 (10.3)	
Falling direction, n (%)				0.001
Forward	66 (4.7)	25 (5.2)	91 (4.8)	
Backward	70 (5.0)	26 (5.4)	96 (5.1)	
Left sideward	522 (37.0)	203 (42.0)	725 (38.3)	
Right sideward	565 (40.1)	203 (42.0)	768 (40.6)	
Forward & left sideward	40 (2.8)	8 (1.7)	48 (2.5)	
Forward & right sideward	36 (2.6)	7 (1.4)	43 (2.3)	
Backward & left sideward	56 (4.0)	5 (1.0)	61 (3.2)	
Backward & right sideward	54 (3.8)	6 (1.2)	60 (3.2)	

## Discussion

This study, for the first time to authors’ knowledge, comprehensively described the characteristics of falls among Chinese older people with hip fracture. We found most fall-related hip fracture patients were in the oldest old age group. Most of them lived in urban areas. The proportion of female patients was more than male patients, with vast majority of falls occurred during the day and at home. The main cause of falling was lost of balance while walking.

In this study, more than half of the patients were over 80 years of age. The risk of falls increases with age because age is associated with impaired balance, reduced mobility, and decreased vision and cognitive abilities [6]. Previous studies in China have shown that older adults were more likely to have unintentional fall-related injuries due to physical and health factors, with the highest rate of moderate-to-severe injuries (e.g., hip fractures) occurring [21]. Most high-income countries, such as Australia and Sweden, have a slightly stable or even decreasing trend in the overall incidence of fall-related hip fractures [14, 15]. Our study found a higher incidence in females than males. There was a similar pattern identified in studies from Cassell and Nilson [14, 15]. This can be potentially explained by females having a higher incidence of osteoporosis and more likely to fracture after a fall [22]. Moreover, frailty is prevalent in the older female population, which may lead to more fall-related injuries [23].

The fact that the majority of hip fracture patients lived in urban areas does not necessarily indicate that the number of patients in rural areas was less than in urban areas. This can be attributed to confounders, such as the availability of the transportation, the family income level and the distance to the study hospitals. Despite more patients living in urban areas from this study, older people living in rural areas are more likely to be vulnerable suffering from a fall and fracture. For nearly two decades, most interventional studies for falls prevention in China have implemented in urban areas, with none studies conducted in rural areas [24]. A cross-sectional study conducted in rural areas of Hubei Province, China, found that rural older adults may have a higher incidence of falls than those living in cities. But unlike this study, they found that rural older adults were more likely to have falls outdoors, which could be related to the fact that older adults in rural areas work outside more often [25].

Consistent with previous research, [21, 26] in this study, patients’ falls mainly occurred during the daytime, and the peak time was between 06:01 and 12:00. This can be due to the fact that older adults are most active at daytime, when family members are not staying at home. More attention should be paid to the characteristics and related factors of falls in the older people during the day, especially in the morning. It is worth noting that more than 50% of the patients had fallen due to imbalance and the most common place for patients to fall was at home, which may be related to older adults spending more time at home without exercise [27]. “Accidents” (including accidental imbalances, slips, and trips) are one of the most common causes of falls in the older people [28]. With age, they will spend less time outdoors and be more likely to fall due to weakness caused by impaired core muscle strength and environmental hazards such as unsuitable steps, escalators, and lack of non-slip flooring [1, 29]. The phenomenon has been particularly prominent since the outbreak when people rarely leave their homes. Some studies have found that the older people are prone to falls when walking, [30] which is consistent with the results of this study, and the use of assistive devices such as walkers and senior shoes can better prevent falls in the older people [31, 32]. In addition, the patient’s tendency to fall during toileting suggests that when the body’s centre of gravity changes spatially, a lack of corresponding muscle strength may trigger an imbalance that could lead to a fall.

There is extremely scarce evidence from the literature that documented the characteristics of falls among the older hip fracture patients. In addition, our previous systematic review also suggested the existing fall-prevention interventions in China were limited and in low quality [24]. Not surprisingly, there was only one published study from China that described the location and timing of falls in 635 patients with fall-related hip fractures. This study characterised falls in nearly 2,000 clinically confirmed hip fracture patients from multiple hospitals. Although this study was designed to address an epidemiological research question, there are important underlying clinical implications. Through understanding the characteristics of falls among the older hip fracture patients, the study results have the potential to support the future development of primary and/or secondary fall-prevention programs.

There are a few limitations of this study. First, study participants were limited to six hospitals in Beijing. Researchers need to be cautious when extrapolating the findings from this study. Second, fall-related information is self-reported data, which might induce recall bias. Lastly, given the main trial was designed as quasi-experimental study without randomisation, there was potential selection bias.

The incidence of fall-related hip fractures in China is currently unknown. There might expect a substantial increase in fall-related hip fractures as ageing accelerates and the incidence of falls increases [13]. It is certain that, as the older population grows, fall prevention for the older people will become particularly challenging. Based on this, we have made several recommendations for future research. First, more research is needed on the characteristics of falls among older hip fracture patients, which can directly inform how to prevent falls and thus reduce the incidence of hip fractures among older people. Second, we urge more research focusing on fall prevention and fall-related hip fracture for older people, particularly for those residing in rural areas, who might be extremely vulnerable. Third, more robust evidence is needed on the biological, behaviour, environmental and socioeconomic risk factors of falls to better understand the epidemiology and develop appropriate context-tailored interventions. Finally, future falls prevention interventions may focus on the strength and balance training, which is evident from the current literature as an effective intervention to prevent falls [33].

## Conclusions

More than half of the patients with fall-related hip fractures were over 80 years of age, and there were more female patients than males. We expect a substantial increase in fall-related hip fractures as ageing accelerates and the incidence of falls increases. Fall prevention for the older people will become particularly challenging. These highlight the importance of implementing evidence-based, effective, high-quality interventions for older people. This study fills gaps in the characteristics of falls to a certain extent in older Chinese hip fracture patients, provides directions for future development of fall-prevention interventions and supports clinical management. But more studies with larger sample sizes and diverse populations are needed to determine fall-related risk factors amongst older Chinese hip fracture patients robustly. Furthermore, as a country with the largest number of older people, this study may provide valuable evidence for other countries facing a similar challenge.

## Data Availability

The data that support the findings of this study are available from Beijing Jishuitan Hospital but restrictions apply to the availability of these data, which were used under license for the current study, and so are not publicly available. Data are however available from the corresponding authors upon reasonable request and with permission of Beijing Jishuitan Hospital.

## Abbreviations

SPSS: Statistical Product and Service Solutions
STROBE: STrengthening the Reporting of OBservational studies in Epidemiology
